# H-Ras gene takes part to the host immune response to COVID-19

**DOI:** 10.1038/s41420-021-00541-w

**Published:** 2021-06-25

**Authors:** Salvatore Sciacchitano, Andrea Sacconi, Claudia De Vitis, Giovanni Blandino, Giulia Piaggio, Valentina Salvati, Christian Napoli, Paolo Marchetti, Beatrice Salimbeni Taurelli, Flaminia Coluzzi, Monica Rocco, Andrea Vecchione, Paolo Anibaldi, Adriano Marcolongo, Gennaro Ciliberto, Rita Mancini, Carlo Capalbo

**Affiliations:** 1 Department of Clinical and Molecular Medicine, Sapienza University of Rome, via di Grottarossa 1035-1039, 00189 Rome, Italy; 2 Laboratory of Biomedical Research, Niccolò Cusano University Foundation, Rome, Italy; 3 UOSD Clinical Trial Center, Biostatistics and Bioinformatics, IRCCS Regina Elena National Cancer Institute, Rome, Italy; 4 UOSD SAFU, Department of Research, Diagnosis and Innovative Technologies, IRCCS-Regina Elena National Cancer Institute, Roma, Italy; 5 Department of Medical Surgical Sciences and Translational Medicine, Sapienza University of Rome, via di Grottarossa 1035/1039, 00189 Rome, Italy; 6 Medical Oncology Sant’Andrea Hospital, University La Sapienza, via di Grottarossa 1035-1039, 00189 Rome, Italy; 7 Unit of Anesthesia, Intensive Care and Pain Medicine, Sant’Andrea University Hospital, Rome, Italy; 8 Medical Direction Sant’Andrea Hospital, University La Sapienza, 00189 Rome, Italy; 9 General Direction Sant’Andrea Hospital, University La Sapienza, 00189 Rome, Italy; 10 Scientific Directorate, IRCSS Regina Elena National Cancer Institute, 00144 Rome, Italy; 11 Department of Molecular Medicine, University La Sapienza, 00189 Rome, Italy

**Keywords:** Viral infection, Predictive markers

## Abstract

Ras gene family members play a relevant role in cancer, especially when they are mutated. However, they may play additional roles in other conditions beside cancer. We performed gene expression analysis using the NanoString PanCancer IO 360 panel in the peripheral blood mononuclear cell (PBMC) of six COVID-19 patients and we found that H-Ras gene was significantly upregulated, while both K-Ras and N-Ras genes were downregulated. In particular, H-Ras gene upregulation was more evident in COVID-19 patients with a more severe disease. We compared our results with those obtained by analyzing two different and independent datasets, including a total of 53 COVID-19 patients, in which the gene expression analysis was performed using the Immunology_V2 panel. Comparative analysis of the H-Ras gene expression in these patients confirmed our preliminary results. In both of them, in fact, we were able to confirm the upregulation of the expression of the H-Ras gene. The exact role of this specific upregulation of the H-Ras gene in response to SARS-CoV-2 infection and its possible role in cancer still remains to be elucidated. In conclusion, H-Ras gene participates to the host immune response to SARS-CoV-2 virus infection, especially in patients affected by the most severe form of the COVID-19.

## Introduction

Growth factor receptor (GFR) signaling besides playing an important role in cancer pathogenesis is also crucial for some virus infections [1–3]. GFRs activate various intracellular signaling pathways, including the one mediated by RAS and RAF. The Ras gene family members encode for four different, highly related protein isoforms (HRAS, N-RAS, K-RAS4A, and K-RAS4B) that are almost ubiquitously expressed in all cell lineages and organs, with some quantitative and qualitative differences of their expression. The Ras gene products are small GTP-binding proteins that play a critical role in the control of basic eukaryotic cell functions, such as proliferation, survival, and differentiation. In addition, they might play specific or overlapping functional roles in many physiological and pathological processes beside cancer, including inflammation.

It has been recently reported that SARS-CoV-2 infection is able to induce a strong up- and downregulation of components of many cellular signaling pathways involved in cancer, including the RAS-RAF/MEK/ERK signaling pathway [4]. Therefore, it appears likely that the activation of this signaling pathway could be involved in the SARS-CoV-2 virus infection and survival too. In line with this, inhibition of Ras signaling pathway by drugs has been suggested as potential antiviral treatment of COVID-19 [5].

Moreover, recent reported cases suggest that COVID-19 may accelerate the course of malignant hematological diseases and induce or exacerbate autoreactive hematopoietic disease [6]. In particular, the temporal relationship of the events may suggest a potential causal relationship between the immunological host response to SARS-CoV-2 infection and the hematopoietic disorder.

No data has been reported in the literature so far regarding the possible role of Ras family genes deregulation, and specifically, of H-Ras gene in COVID-19. In the present study, we analyzed the Ras family gene expression levels in peripheral blood mononuclear cell (PBMC) of COVID-19 patients.

## Methods

Total RNA was extracted and purified from PBMC isolated from eight blood samples, six from of COVID-19 patients, subdivided in mild/moderate (*n* = 2) and severe/critical (*n* = 4), according to WHO criteria and two from healthy controls (HC). Gene expression was measured with the NanoString PanCancer IO 360 panel using as input 30–100 ng total RNA of each sample, as previously reported [7, 8] and according to the manufacturer’s instructions. After the Codeset hybridization the samples were analysed with the nCounter Digital Analyzer. RCC files were analyzed using nSolver analysis software (Version 4.0) following the manufacturer’s protocols. Negative and positive controls were included in probe sets and used for background thresholding and for normalization. Normalized counts were further analyzed by MATLAB R2020b. Gene expression differences were evaluated by a Wilcoxon rank sum test for two groups and Kruskal-Wallis test for more than two groups. Same statistical tests were used to asses gene expression differences in two validation cohorts, analyzed using the NanoString Immunology_V2 panel [9, 10] (Table 1).

**Table 1:** Comparative *Ras* genes expression in three independent dataset of COVID-19 patients analyzed by NanoString nCounter technology.

NanoString nCounter human gene expression panels	Genes included	COVID-19 patients			Healthy controls	Reference
		Mild to moderate^a^	Severe to critical^a^	Total		
PanCancer IO 360	770	2	4	6	2	Present study
Immunology_V2	594	10	21	31	13	Hadjadj et al. [9]
Immunology_V2	594	N/A	N/A	22	10	Vastrad et al. [10]
Total		12	25	59	25	

^a^World Health Organization (WHO) COVID-19 severity criteria.

## Results

**Fig. 1:**
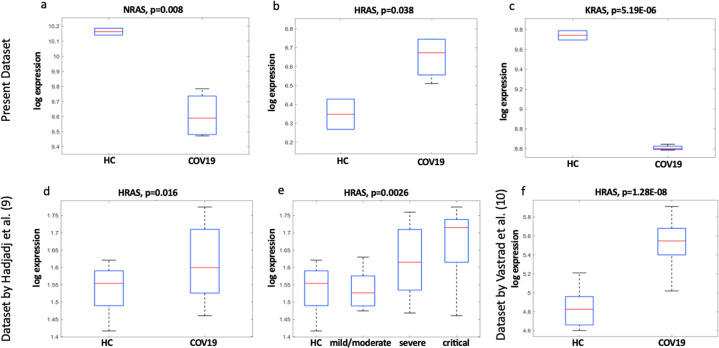
*Ras* genes expression in COVID-19 patients. The expression of H-*Ras* genes is reported in log-expression units for the three different dataset (**b**, **d**, **e**, **f**). Data regarding *N-* and *K-Ras* are available only for our dataset and were not present in the immunology panels used for the analysis of the other datasets (**a**, **c**). Statistically significant differences in the expression of *Ras* genes between healthy controls (HC) and COVID-19 patients (COV19) are reported.

Dysregulated Ras family gene expressions represent a common event in our samples obtained from COVID-19 patients, compared to HC. This feature is even more evident when we consider severe/critical disease. In particular, COVID-19 patients with a more severe disease showed a significant upregulation of H-Ras and a coexistent downregulation of both K-Ras and N-Ras gene expressions when compared to control cases (Fig. 1a–c). In order to verify these results, we retrieved and analyzed data of gene expression signatures from two large cohorts of COVID-19 blood samples. In particular, by reanalyzing one published independent large dataset [9] we confirmed that H-Ras was indeed expressed at a higher level in COVID-19 patients compared to control ones and that its expression was correlated also with the disease severity in a statistically significant manner (Fig. 1d, e). To obtain another confirmation of such results, we analyzed another large microarray dataset of COVID-19 patients [10]. A significant H-Ras upregulation compared to control ones was, again, observed also in this study (Fig. 1f). Taken together, results of our study and the re-analysis of data from two large and independent case series, indicates that H-Ras gene expression is significantly increased in PBMC isolated from blood of COVID-19 patients and its increased expression correlates with the disease severity.

## Discussion

Dysregulation of RAS cycling can promote human disease conditions, with somatic mutations in the Ras genes being prominent drivers of cancerogenesis and Ras germline mutations contributing to a group of related developmental disorders known as the RASopathies [11]. Despite being one of the most frequently mutated signaling pathways in human cancer, various aspects of RAS biology are still poorly understood. In particular, dysregulated RAS signaling affects directly not only the development of various pathological condition in infected cells but also in host physiological processes, such as immunological response to viremic condition and inflammation [12]. Our preliminary observation, confirmed by bioinformatics analysis of two large independent datasets, indicates that H-Ras overexpression in PBMCs is part of the immune response to COVID-19, especially in patients with severe disease conditions. On the contrary, K-Ras and N-Ras, showed an opposite behavior and are both downregulated. It is attractive to speculate that H-Ras overexpression, triggered by viral infection, might act as a stimulator of precancerous conditions or of cancer stem cells compartment and that could represent one of the links connecting viral infection to cancer. A crescent number of studies reported that patients with malignant hematological diseases are at increased risk of complications from SARS-CoV-2 not only due to immune compromise related to the malignancy but also due to the host immunologic and cytokine response to SARS-CoV-2 infection [6]. We hypothesize that, due to the specific interaction between SARS-CoV-2 virus and infected patients, a cascade of out-of-control events can ensue determining some unexpected pathologic conditions such as the progression, reactivation of hematological malignancies or the development of proliferative disorders. It would be interesting to elucidate whether H-Ras gene overexpression that we observed in the PBMC of COVID-19 patients is part of a specific response to SARS-CoV-2 infection or it occurs also with other viruses.

In conclusion, we report here that the H-Ras gene the is overexpressed in the PBMC of COVID-19 patients, especially in those presenting a more severe condition. Such overexpression represents a molecular event activated by the immune cells upon SARS-CoV-2 infection. Our observation paves the way to a new area of research focused the role of Ras family genes in the host immune response to COVID-19 as well as to other viral infectious diseases.
